# Isolation and molecular identification of mycoplasma genitalium from the secretion of genital tract in infertile male and female

**Published:** 2014-09

**Authors:** Naeime Mohseni Moghadam, Babak Kheirkhah, Toraj Reza Mirshekari, Majid Fasihi Harandi, Elham Tafsiri

**Affiliations:** 1 *Department of IVF, Afzalipoor Hospital, IVF Center, Kerman, Iran.*; 2 *Department of Microbiology, College of Science, Kerman Branch, Islamic Azad University, Kerman, Iran.*; 3 *Department of Pathology, Afzalipoor Medical College, Kerman, Iran.*; 4 *Department of Parasitology, Afzalipoor Medical College, Kerman, Iran.*; 5 *Department of Molecular Medical Genetic, Biotechnology Research Center, Pasteur Institute, Tehran, Iran.*

**Keywords:** *Mycoplasma genitalium*, *polymerase chain reaction*, *Asymptomatic Infections*, *Infertility*, *Sequence Analysis*

## Abstract

**Background:** Mycoplasmas can cause acute and chronic diseases at multiple sites with wide-range complications and have been implicated as cofactors in diseases. The infections influenced form genital mycoplasmas specifically *Mycoplasma hominis* and *Mycoplasma genitalium* potentially affect reproductive disorders, and infertility.

**Objective:** Isolation and molecular identification of *Mycoplasma genitalium* from the genital tract of infertile male and vaginal discharge of infertile female referred to Infertility Center of Kerman in 2013.

**Materials and Methods: **This study was a randomized, prospective study. We included 100 infertile male and 100 infertile female that were referred to the Infertility Center of Kerman. Then for isolation and molecular identification of *Mycoplasma genitalium* from urethral and vaginal discharge polymerase chain reaction was performed on *Mycoplasma genus* and *genitalium.*

**Results: **From a total of 100 semen samples 45 patients (45%) were mycoplasma-positive and 13 (28.8%) were genitalium species positive. Also, from a total of 100 women samples 43 women (43%) were mycoplasma-positive and 10 (23.2%) were genitalium species positive. Positive samples were sequenced and phylogenetic tree was drawn.

**Conclusion:** According to the results of this study, a high percentage of infertile male and female were infected with the *Mycoplasma genitalium*. For prevention of harmful and significant consequences of this infection, we suggest a screening program in symptomatic infertile couples.

## Introduction

*Mycoplasma genitalium *is a member of genital mycoplasmas, which is emerging as an important causative agent of sexually transmitted infections both in males and females. Mycoplasma bacteria are tiny parasites that contain 580070 base pairs in whole genome. *Mycoplasma genitalium, the* smallest known genome of any free-living organism, lives in the ciliated epithelial cells of reproductive and respiratory system in mammalian (1). These small sized organisms need vitamins, amino acids, nucleic acid precursors and especially lipids for growth; however, there is no cell wall in this species. They are often called mycoplasmas which constitute the class of Mollicutes and they do not produce peptidoglycan precursors. 

*Mycoplasma genitalium* genome consists of 528 genes (482 protein coding gene) which all of them are located on one chromosome. These organisms were isolated in1980 from two men with non-gonococcal infection. *Mycoplasma* *genitalium* infects one in fifth infertile couples (2). Prevalence of *Mycoplasma genitalium*, in infertile populations in different areas in developed countries is estimated from 39% to 85%. The incidence of infertility in Black people is 1.5 times higher than that of white people. This organism affects the number, shape, maturation and motility of sperm (3-      6  ). 

Mainly abnormal sperm parameters (count, morphology, progressive motility and viability), presence of anti-sperm antibodies and obstruction of genital tract affect men fertility’s rate temporarily or permanently (7). Genital mycoplasmas infection potentially affects reproductive disorders, and infant mortalities. Therefore, detection of these organisms is an important issue that should be considered and adequate diagnostic methods should be used to identify these organisms. 

In the female genital tract, infection can include different parts of the cervix, endometrium and fallopian tube. The extent of this infection in different diseases and its pathogenesis might be related to anatomic site of involvement. Some infections can lead to infertility in both men and women. Sometimes it might cause disruption of embryo implantation in the uterus and results miscarriages. In men infection of the prostate gland or the semen may influence on sperm through certain ways. Infected cells are able to reduce sperm swimming ability. Some infections can cause blockage of the sperm in the male reproductive tract, leading to the cessation of sperm transfer in IVF procedures (8). 

Recently many investigations have been focused on the detection of mycoplasma agents from male and female genital tracts, via Polymerase chain reaction and molecular studies of discovered bacteria. However there is no probe on the molecular characterization of these bacteria in Iran. The aim of this study was molecular identification of Mycoplasma genitalium from the genital tract of infertile male and vaginal discharge of infertile female referred to the Infertility Center of Kerman in 2013.

## Materials and methods

This randomized, prospective study was done on 100 infertile men and 100 infertile women, with purposive sampling during five months, which referred to Infertility Center of Kerman in 2013. All of referred patients passed through traditional clinical examinations and routine tests of fertility and infertility (Hormonal Testing such as: LH, FSH, prolactin, testosterone), laparoscopy, spermogram, and the results were confirmed by urologist and gynecologist. Also, this group had sexual abstinence for 48 hr, A couple after a year of regulation intercourse without prevention was defined as infertile. 

Therefore, 100 infertile men were identified and selected. Semen samples were collected after a three days abstinence period, by masturbation. Patients had not taken any antibiotic since one week before collecting the semen sample. Moreover, before collecting the sample, patients had to wash their hands and genital area with water and soap. Samples were collected in sterile plastic containers which were used for collection of urine. Immediately after collection, the semen samples were placed in incubator and allowed to liquefy at 37^o^C for up to 30 min before commence of the analysis. Semen analysis was performed according the WHO guidelines (9). 

A typical semen analysis measures the following parameters: Volume and viscosity of semen, sperm cell concentration (density), total number of sperm cells, sperm motility (percentage of moving sperm and the way the sperms moved), number of normal and non-normal (damaged) sperms (morphology), coagulation and liquefaction, PH (acidity), number of immature sperm cells, leucocytes (white blood cells) and number of infected cells. In other hand, vaginal discharge samples of 100 infertile women were collected after preliminary examinations and infertility test such as hormone test, sonography of the uterus and ovaries, then the infertility was confirmed by gynecologist. 

The vaginal discharge was obtained by speculum and one sterile cotton swab was used to collect specimens. The vaginal swab samples dissolved in Phosphate buffered saline (PBS) (1.5 mM KH_2_PO_4_, 10 Mm NA_2_HPO_4_, 2.5 Mm KCL, 0.1 M NACL and pH= 7.4 was solved), semen samples and vaginal discharge were stored at -20^o^C and -70^o^C, respectively. Consequently, DNA was extracted according to manufacturer’s manual (Sina clone, Cat No: 881613) and stored at -20^o^C. All clinical samples were tested by PCR assay for the presence of *Mycoplasma genitalium*. Amplification of the 16S rRNA gene was performed with a pair of primers (Table I) complementary to the regions close to the 5́ and the 3́ termini of the gene. 

The DNA from each sample was subjected to two PCR, one for detection of mycoplasma strain and the other one was PCR-positive samples to determine species. According to European Pharmacopoeia 2005 negative and positive controls were PPLO broth media and standard strain of *Mycoplasma genitalium* (strain ATCC 33530/ G-37/ NCTC 10195), respectively. DNA amplification were carried out in a total volume of 25 µl and contained: 12.5 µl Tag DNA polymerase Master mix RED (2X), (Ampliqon, Denmark), 1µl of each primer, 3-4 µl extracted DNA, 7 µl distilled water. 

The thermal-cycling conditions for Mycoplasma strain were as follows: 95^o^C for 6 min, followed by 35 cycles of 94^o^C for 1 min, 55^o^C for 1 min and 70^o^C for one minute. Also, the thermal-cycling condition for *Mycoplasma genitalium* species was as follows: 95^o^C for 4 min, followed by 35 cycles of 94^o^C for 45s, 62^o^C for 1 min and 72^o^C for 2 min. PCR was carried out using two kinds of program able thermal cycler (Primus and Master gradient). Positive and negative controls were included in all tests. 1 µl aliquot of each PCR products was mixed with 2µl loading buffer (6X) and loaded on 1.5% agarose gel which then stained with 0.5 µl/ml ethidium bromide (100 volts for 1 hr) and visualized by UV Translluminator. 

**DNA purification**

The PCR product was amplified and DNA purification was performed by High pure PCR Product Purification Kit column (Macrogen Company Korea) After purification PCR products were sent to Bioneer Company (South Korea) for sequencing. sequenced then the bacterial sequences were aligned through multiple alignments by using Bio Edit software with Clustal W method. On the other hand, nucleotide sequences were compared with each other by using MEGA 5 software. Then similarity matrix was displayed in excel and neighbor-joining tree was analyzed and plotted based on phylogenetic tree with Bootstrap 1000. To ensure the specificity, the sequences were compared with other mycoplasma sequences that are available in the NCBI gene bank. The similarity matrix of this study was designed after comparing them with isolates of other parts of the world that are recorded in NCBI (Figure 3). Then the phylogenetic tree was drawn (Figure 4).

## Results

**Electrophoresis and detection of PCR products**

**Figure 1:**
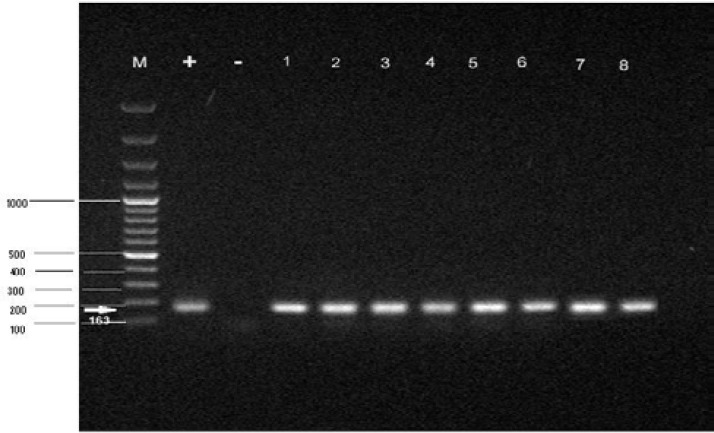
PCR products of Mycoplsma strain on Agarose gel. From left: lane +. Mycoplsma positive control. Lane -. Mycoplsma Negative control.Lane 1-4 positive male samples.Lane5-8 positive female samples

**Figure 2:**
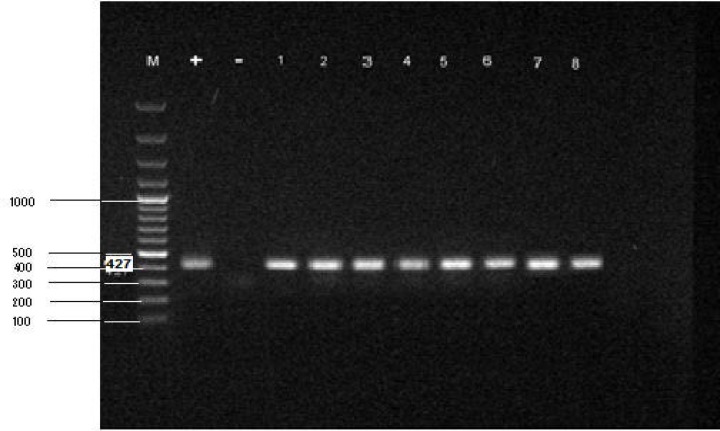
PCR products of *Mycoplasma genitalium* on agarose gel. From left: Lane M. DNA ladder (100bp). Lane +. Positive control. Lane -. Negative control. Lane 1-4 positive male samples (427 bp).Lane 5-8 positive female samples (427bp).

Figure 1 show the bands (163bp) which were obtained from mycolplasma-positive male and female samples. Figure 2 demonstrates the bands (427bp) for *Mycoplasma genitalium*. All of the electrophoresed PCR products were run with positive and negative control.

**Biological survey results**

**Frequency distribution and percentage of positive samples**

In this study in order to isolate *Mycoplasma genitalium* as genital infection among males and females, PCR was used and we did not culture the bacteria because PCR is a specific and quick technique. 200 samples from infertile men and women were taken, from which 88 cases (44%) were confirmed for mycoplasma species and 23 (14.26%) of patients were genitalium. And abundance of species based on the specimens is shown in Table II. Evaluation of semen parameters in samples showed: motility of sperm samples from 0-45%, 10% of samples with zero mobility (immotile). Normal morphology of the samples was 11.8% and 25% of those with abnormal morphology were between 0-5. 

The concentration of sperm per ml of semen samples from 0-98 million. Semen volume in the samples was 0.5-8 ml. The minimum and maximum age was 21 and 65, respectively. Round cell (RC) per milliliter was 0-30 million in the samples. Rapid movement of the sperm ranged from 0-15%. Motility sperm quantity was 0-70%. Sperm motility was between 0-100%. In women’s group from 20-45 years old and the range of infertility duration was from 1-18 years.

**Statistical analysis**

Data analysis was done using crosstabs and Chi-square test for qualitative variables and comparison of study variables was performed by SPSS software (Statistical Package for the Social Sciences, version 20.0, SPSS Inc, Chicago, Illinois, USA)The p<0.05 was considered to be statistically significant. Comparison of the parameters of the standard semen analysis showed that the presence of *Mycoplasma genitalium* in semen samples is associated with low sperm concentration (p=0.001), motility (p=0.002), abnormal sperm morphology (p=0.000), sperm fast motility (p=0.004) and age (p=0.001). The mean values of semen volume, duration of infertility, sperm viability and leukocyte count were not significantly related to the detection of genital *Mycoplasma genitalium* in semen specimens.

**Table I:** Nucleotide sequences and primers used for identification of *M. genitalium* by PCR

**Primer**	**Target gene**	**Sequence**	**Length (bp)**	**Reference**
GSO	16S rRNA	F: 5^/^-GGGAGCAAACAGGATTAGATACCCT -3^/^	163	(10)
MGSO	16S rRNA	R: 5/-TGCACCATCTGTCACTCTGTTAACCTC-3/	163	(10)
45F	16S rRNA	F: 5/-TACATGCAAGTCGATCGGAAGTAGC-3/	427	(11)
447R	16S rRNA	R: 5/-AAACTCCAGCCATTGCCTGCTAG-3/	427	(11)

**Table II:** Frequency distribution and percentage of positive samples obtained based on studied Genus and species

	**Positive (Mycoplasma)**		**Positive (** **Mycoplasma** ** genitalium)**		**Total positive**
	**Percent**	**frequency**	**percent**	**frequency**	**frequency**
Male	45	45	28.8	13	100
Female	43	43	23.2	10	100
Total	44	88	26.14	23	200

**Table III:** Profile of M. genitalium selected from the world (Gene bank) for phylogenetic analysis. The sequences were compared with other mycoplasma sequences that are available in the NCBI gene bank

**Country**	**Accessibility**
USA	NR_074611
USA	NR_074554
USA	CP000925
USA	CP003770
USA	CP003771
USA	CP003772
USA	CP003773
Denmark	AY466443
USA	L43967
Denmark	NR_026155

**Figure 3:**
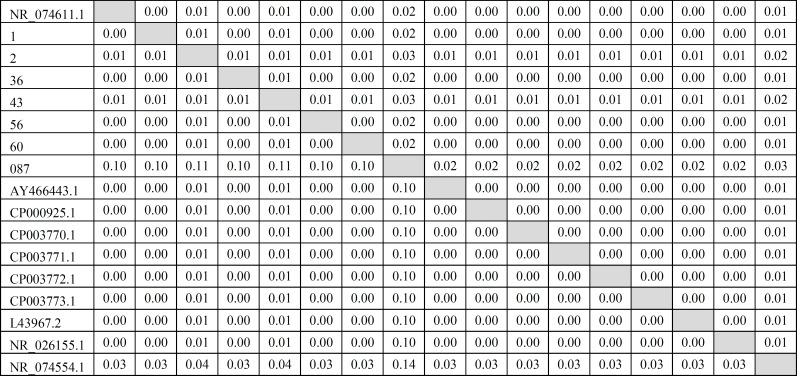
The similarity matrix of this study was compared with isolates of other parts of the world that are recorded in NCBI.

Sequence alignment was done with (Clustal W) BioEdit software.

**Figure 4:**
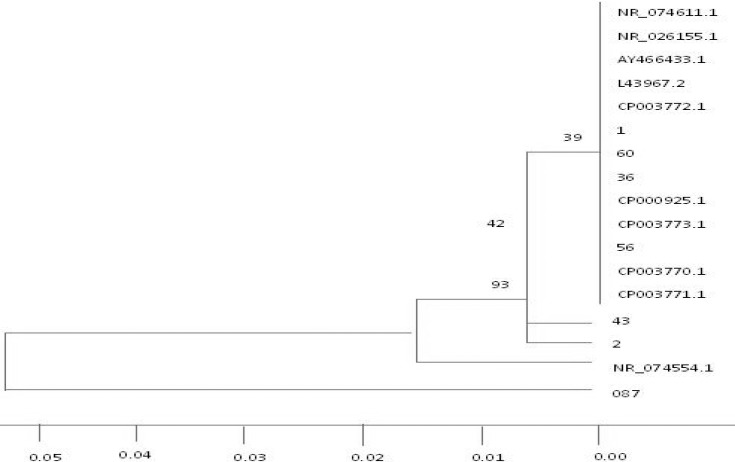
Phylogenetic tree of seven strains of *Mycoplasma genitalium* strains isolated in this study and other strains of this bacterium in Gene bank. phylogenetic tree was drawn with Bootstrap 1000.

## Discussion

This research illustrates the significant of *Mycoplasma genitalium* as a pathogen. In additional, its genome has been extensively studied in order to determine the minimal set of essential genes for life (13). Genital infections have main role in male infertility as risk factors. Bacteria or their toxins bind to the sperm cause immune system stimulation and damage to spermatozoa in all areas of urinary and reproductive tract. Most of infertile men have positive history of STDs (sexually transmitted diseases) as gonococcal or non-gonococcal urethritis. Moreover the asymptomatic infected men usually will infect their wives and cause secondary couple infertility problems. 

Most of infertile men, wives have asymptomatic STDs which may be related to their infected husbands. It is approved that the infected semen has effects on fertilization success rate in ART. The sperm in infected ejaculation might cause contamination of egg or embryo (14). *Mycoplasma genitalium *is a common causative agent of sexually transmitted infections in men and women. With the advent of PCR and other molecular methods for detection of *Mycoplasma genitalium* appropriate studies are possible, and even it is easily can be isolated after birth*. Mycoplasma genitalium* currently is one of the most distinguished agents from men who are infected by nongonococcal infection*. M. genitalium* in pelvic inflammatory disease and infertility is a common complication among infected people (15). 

Also, studies have shown the directly relation of *Mycoplasma genitalium* to infection of the cervix and vagina in women. These bacteria can cause pelvic inflammatory disease and infertility, infections of this organism is the upside potential*. Mycoplasma genitalium* is associated with some cases of acute and chronic non-gonococcal urethral infection. This organism is directly associated with cervical infections, inflammation of the uterus and pelvic inflammatory disease and genital mycoplasmas are the types that cause genital-urinary infections (16,17). 

Among 23 strains of *Mycoplasma genitalium* which was confirmed by PCR, 7 isolates was sent to pioneered company for 16s rRNA gene sequencing. Sequence alignment aligned and comparison by using mentioned software showed that the 7 separated *Mycoplasma genitalium* were divided into four lineages. Phylogenetic tree which was drawn based on alignment confirmed this finding. The first dynasty was located in 087, the second in 2, the third in 43 and the fourth in 56, 36, 60 and 1 isolates, respectively, in the same phylogenetic branch (Figure 4, Table III). 

In this study for the first time in Iran mycoplasma infection among males and females were considered phylogenetically. By identifying mycoplasma might help us in understanding its distribution and epidemiology. PCR is a molecular and sensitive technique in comparison to other diagnostic tests (10). Ahmadi *et al* in their study confirmed this technique as a validated one in detection and identification of *Mycoplasma genitalium* (1). PCR test can be used as a reliable method for the isolation of genital mycoplasmas (20). Vosoughi showed high sensitivity and specificity of the PCR method in comparison with culture method by working on *Mycoplasma huminis* in infertile males (8). And because it can be a quick and efficient diagnostic test for male infertility, so it may be used in the Infertility Centers (18). In our study among 200 cases of infertile and mycoplasma infected male and female, 88 patients (44%) were mycoplasma positive , therefore, we can conclude that genital mycoplasma plays an important role in the causes of such infections among men and women (19). 

Bakhshande Nosrat *et al* reported the rate of mycoplasma in patients with urinary tract infection was 53% which was in line with our findings (12). It looks like that the role of mycoplasma infection is higher in patients with infertility. In a survey by Stellrech *et al* in 2004 in America were 21 infertile men and women were analyzed by PCR, 17 of whom were positive for mycoplasma contamination (21). The results also showed that infection with mycoplasma genus and species genitaliumin men is more than women, however, there was not a significant difference in hominis genus, so we can conclude that mycoplasma infection is not related to its genus. The present results suggest that the intensity of the urinary mycoplasma in women is more than men in Kerman city. Since, the overall rate of urinary tract infections in women is higher than in men. The reason might be because of the anatomical position of the urinary tract (18).

In this study it was observed that none of the infected semen samples which were mycoplasma PCR positive had azoospermia. These findings support that *Mycoplasma genitalium* infection and inflammation is bound to the sperm cells. This bacterial infection cannot be passed when there are no sperm cells. In fact, the results were similar to other studies which carried out (8). According to Mousavian *et al* research on isolation and determination of the prevalence of mycoplasma infections in patients who were referred to Imam Khomeini Hospital, Ahvaz, among 221 samples, 121 patients had urogenital infected (54.7%) and 100 patients had respiratory tract infection (45.3%). Among respiratory tract infected patients, 48% were male and 52% were female, also, in urinary tract infected cases 31.4% were male and 68.6% were female. The results of this study showed that the risk of mycoplasma infections in men were more than women (22). 

The results showed that from a total of 88 patients, 23 were infected with genitalium (14.26%) and 65 patients (86.73%) were infected to other species or possibly *Mycoplasma hominis* or *Ureaplasmalyticum*. *Mycoplasma genitalium* which was isolated from patients with genital infection in Kerman based on genetic similarity belonged to four quite distinct lineages. Bacterial isolates and all four lines had very little genetic similarity. Isolates 56, 36, 60 and 1 in a gene bank in Denmark and America are separated geographically and have high genetic similarity, so they are placed in a line. 

In fact, it can be recorded as a native strain in the gene bank. Strains 2 and 34 have been isolated in the two lineages, although little similarity with other isolates and isolates of the present study are available in the Gene bank. None of the isolates with an isolate in Denmark and other countries have similar genetic lineages, so they are completely independent, perhaps this is due to certain factors including, special weather conditions and climate in Denmark. 

## Conclusion

In conclusion *Mycoplasma genitalium* was the most important bacterial agent who was isolated from patients with genital infections was in Kerman. Despite various studies carried out in Iran and the other parts of the world regarding mycoplasma in urinary-reproductive tract, nucleotide sequencing and phylogenetic analysis, Mycoplasma as a cause of urinary-genital tract infections has not been done in Iran yet. Thus, according to the differences in various strains of bacteria in pathogenesis and their interest to various tissues, molecular analysis and heterogeneity studies seem to be necessary. Furthermore, the results of this study can help us in treatment of this disease. Also, Mycoplasma isolates with determined identity is available for research in future, so it can provide a new pathway to detection of other infections in human reproductive system.

Since urinary tract infections can be easily lead to genital infections and previous studies have shown the genital mycoplasma infections is caused to infertility in men and women, so the urinary tract of Mycoplasma infections is strongly recommended to be taken seriously (15). Therefore, in the case of genital mycoplasma infection, couples should be treated with appropriate antibiotics. Furthermore, attempting to determine the prevalence and molecular identity requires an independent study on investigation of the relation between these two infections.

## Footnote

***This article extracted from M.Sc. thesis. (Naeime Mohseni Moghadam)***
